# Effectiveness of shengxuexiaoban capsules combined with glucocorticoid therapy for immune thrombocytopenia: A meta-analysis

**DOI:** 10.1371/journal.pone.0275122

**Published:** 2022-09-30

**Authors:** Meng-Yuan Ding, Bing Li, Meng Yang, Wen-Sheng Zhai, Chun-Dong Song, Jian Zhang, Qiu-Yue Zhang, Peng-Fei Li, Li-Ya Liu

**Affiliations:** 1 College of Pediatrics, Henan University of Chinese Medicine, Zhengzhou, Henan, China; 2 The First Affiliated Hospital of Henan University of Traditional Chinese Medicine, Zhengzhou, Henan, China; Qatar University, QATAR

## Abstract

**Objective:**

To evaluate the effectiveness of the Shengxuexiaoban Capsules combined with glucocorticoid therapy for immune thrombocytopenia (ITP).

**Methods:**

We collected randomized controlled trials (RCTs) using shengxuexiaoban capsules in combination with glucocorticoid to treat ITP by searching major Chinese and English electronic databases. The outcome indicators were the effective rate, recurrence rate, the number of platelets in the blood, recovery time of platelets, and adverse reactions. We used STATA 16.0 and RevMan 5.3 for meta-analysis and GRADE pro. for evidence quality evaluation.

**Results:**

A total of 27 RCTs were included in the meta-analysis, and the results showed a significant difference (all p<0.05) in the effective rate, recurrence rate, the number of platelets, and the recovery time of platelets (≥ 100×10^9^) between ITP patients in the control group (who received glucocorticoid therapy alone) and test group (who received glucocorticoid therapy combined with the Shengxuexiaoban Capsules). And that Shengxuexiaoban capsules combined with glucocorticoid therapy were safe. The funnel plot and Egger’s test results indicated no obvious publication bias. The GRADE evidence rating showed an intermediate quality of evidence rating for recurrence rate and overall effectiveness.

**Conclusion:**

Glucocorticoid therapy combined with the Shengxuexiaoban Capsules showed more effectiveness in the treatment of ITP. It can improve the effective rate, reduce the recurrence rate, increase the number of platelets and shorten the recovery time of platelets, and has a good safety profile.

## 1. Introduction

Immune thrombocytopenia (ITP) is a common bleeding disorder encountered in clinical practice. Patients with ITP usually have spontaneous skin and mucous membranes bleeding caused by a decrease in the number of platelets in the blood [1]. At present, glucocorticoid remain the first choice for treating of ITP [2, 3], which significantly increases the number of platelets. However, long-term use of glucocorticoid can lead to various adverse effects [4], such as central obesity and osteoporosis. Recent studies have discovered that the Shengxuexiaoban Capsules are effective for ITP. Because it contains Indigo Naturalis, Cortex Moutan, Forsythia, Agrimonia, and Licorice, which can clear away heat and toxic materials, cool blood, arrest bleeding, disperse blood stasis and reduce freckles. However, studies reporting on Shengxuexiaoban Capsules for patients with ITP were all sample sizes studies. Herein, we performed a meta-analysis of the included RCTs to present evidence for the clinical treatment.

## 2 Materials and methods

### 2.1 Inclusioncriteria

#### 2.1.1 Study design

All included studies were RCTs, and the language was not limited, whether blinding or allocation concealment.

#### 2.1.2 Research objects

a. Patients diagnosed with ITP (meeting the clinical diagnostic criteria of primary immune thrombocytopenia).

b. It is not affected by the included patients’ age, sex, and region.

#### 2.1.3 Intervention comparison

The patients in the control group received glucocorticoid therapy alone, while those in the test group received glucocorticoid therapy combined with the Shengxuexiaoban Capsules.

### 2.2 Exclusion criteria

Repeated studies, unrelated articles, animal tests, reviews, conference reports, and studies with unclear data were excluded from the analysis.

### 2.3 Outcome

The outcome indicators were the effective rate (the effective rate = (number of apparent cases + number of good cases + number of improved cases)/total number of cases in the case of consistent clinical efficacy criteria. The other is the effective rate = (number of complete responses + number of effective cases)/total number of cases in the case of consistent clinical efficacy criteria), the recurrence rate, the number of platelets in the blood, and the recovery time of platelets, adverse reactions.

### 2.4 Retrieval of RCT

A full-test search was performed in the databases to select the RCTs where the glucocorticoid therapy was combined with the Shengxuexiaoban Capsules to treat ITP, including China National Knowledge Infrastructure (CNKI), VIP database (VIP), China Biology Medicine disc (CBMdisc), Wanfang Data Knowledge Service Platform (Wanfang Da-ta), PubMed and Embase. The retrieval time was the time from the establishment of the database to April, 2022. The following key search terms and their potential combination were used: "升血小板胶囊 (Shengxuexiaoban Capsules)", 免疫性血小板减少症 (Immune Thrombocytopenia)" and "激素 (glucocorticoid)", and the English keywords included "Immune Thrombocytopenia", "Idiopathic Thrombocytopenia", "shengxuexiaoban Capsules" and "Glucocorticoid". The search mode included combining keywords with free words. Take the China Biology Medicine disc as an example, the search strategy is as follows: #1 Search (("Immune Thrombocytopenia"[Mesh]) OR (Idiopathic Thrombocytopenia purpura) OR Immune Thrombocytopenic Purpura OR Purpura thrombocytopenia; #2 Search (Shengxuexiaoban Capsules); #3 Search (Glucocorticoid) OR Prednisone; #1 AND #2 AND #3.

### 2.5 RCT selection and data extraction

Two researchers followed the inclusion and exclusion criteria and independently selected the RCTs and collected data, including basic information about the selected RCTs (number, gender distribution, and average age of the patients in the control group and test group), specific intervention measures, outcome indicators, result data. In the event of any disagreement, a third researcher was invited.

### 2.6 Quality evaluation

The quality of the included RCTs was evaluated by the risk of bias assessment tool of the Cochrane Handbook for Systematic Reviews of Interventions (Version 5.1.0). The biases mainly covered six aspects: selection bias, performance bias, measurement bias, attrition bias, reporting bias, and other biases, and were categorized with "low risk", "unclear risk" and "high risk" one by one.

### 2.7 Statistical analysis

RevMan5.3 and Stata16.0 software were adopted for the Meta-analysis. The P and I^2^ values were used to assess the heterogeneity. P-value < 0.1 or I^2^ > 50% indicated the presence of heterogeneity, and a random-effect model was applied for heterogeneity and sensitivity analysis. For a P-value > 0.1 or I^2^ < 50%, a fixed-model was applied.

### 2.8 Evidence quality evaluation

The results of the meta-analysis were evaluated using the GRADE method, and considered for degradation in terms of risk of bias, inconsistency, indirectness, accuracy, and publication bias. They were classified as "high quality", "moderate quality", "low quality" and "very low quality".

## 3 Results

### 3.1 RCT selection results

**Fig 1:**
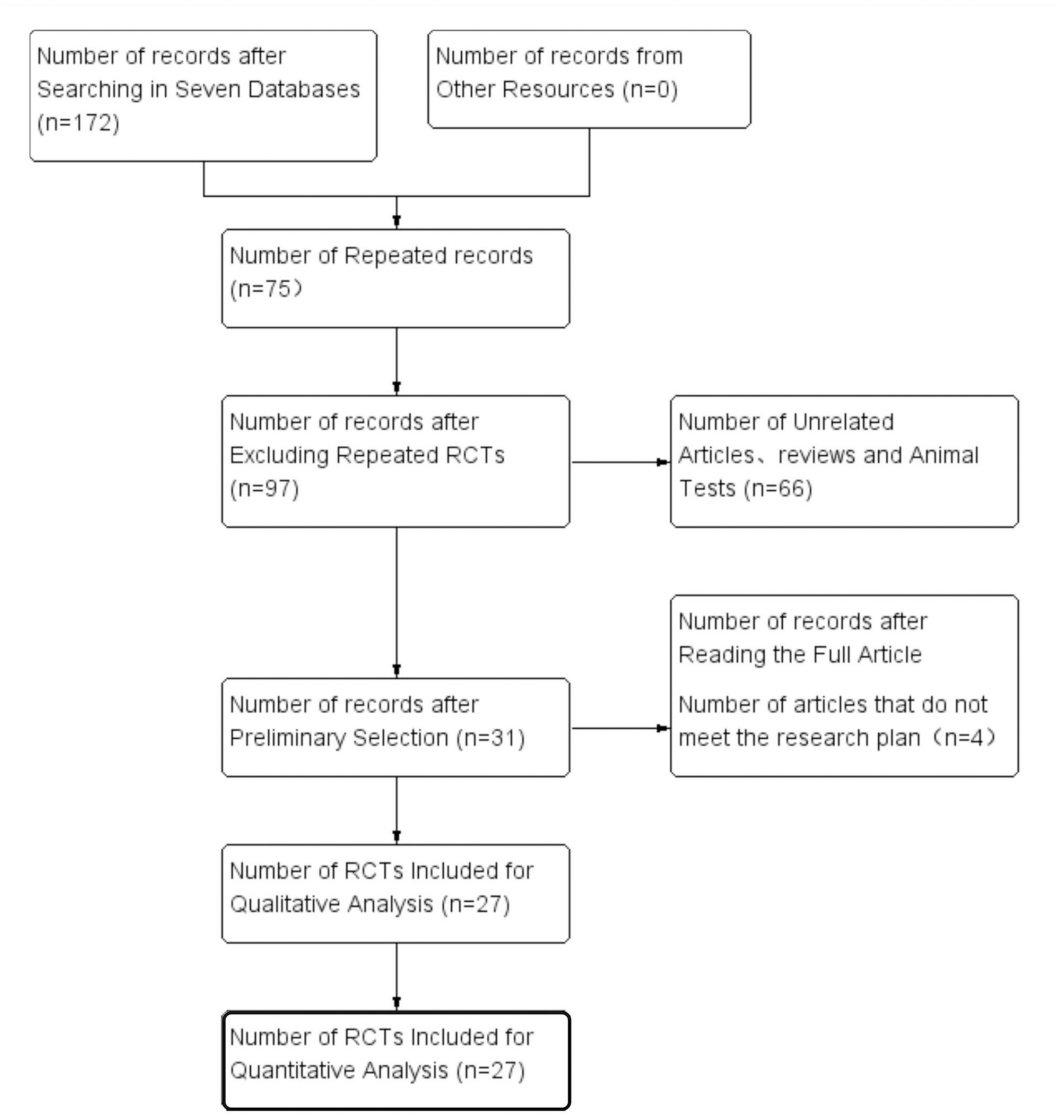
Flow chart of study screening.

According to the search strategy, 172 Chinese pieces of literature were preliminarily selected, and no English literature was found. After excluding 75 pieces of literature with duplicate content, 27 RCTs were finally included in the meta-analysis. The selection process is shown in Fig 1.

### 3.2 Basic characteristics of included subjects

A total of 1,668 patients who were diagnosed with ITP were included in the 27 RCTs. 50.7% were treated by glucocorticoid therapy combined with the Shengxuexiaoban Capsules, and there maining eight hundred twenty-three by glucocorticoid therapy alone. In four RCTs [5–8], the observation time was not reported. In the remaining 23 RCTs, the course of the disease ranged from two weeks to two years. The baseline characteristics of ITP patients (n = 1668) were presented in Table 1.

**Table 1:** Characteristics of randomized controlled trials of shengxuexiaoban capsules in combination with glucocorticoid for ITP.

Study ID	Sample Size (T/C)	Test Group	Control Group	Observation Time	Outcome Indicators
Li Wenlei 2018	15/15	Pednisone+Shengxuexiaoban Capsules	Pednisone	3M	(1) (4) (5)
Hu Tianlian 2016	15/15	Pednisone+Shengxuexiaoban Capsules	Pednisone	12M	(3)
Cai Gangli et al. 2019	30/30	Pednisone+Shengxuexiaoban Capsules	Pednisone	3M	(1)
Yuan Fang 2015	40/40	Pednisone+Shengxuexiaoban Capsules	Pednisone	6M	(1) (5)
Yu Bin 2019	35/30	Pednisone+Shengxuexiaoban Capsules	Pednisone	3M	(1) (3)
Jiang Ming et al. 2021	38/38	Pednisone+Shengxuexiaoban Capsules	Pednisone	7M	(2)
Du Hui 2014	10/10	Pednisone+Shengxuexiaoban Capsules	Pednisone	12M	(1) (3)
Xiang Qi et al. 2015	30/30	Pednisone+Shengxuexiaoban Capsules	Pednisone	3M	(1) (3)
Wang Wei et al. 2009	31/31	Pednisone+Shengxuexiaoban Capsules	Pednisone	3M	(1) (5)
Wang Jiru 2012	43/42	Pednisone+Shengxuexiaoban Capsules	Pednisone	24M	(1) (2) (5)
Chen Jianlan 2019	43/42	Pednisone+Shengxuexiaoban Capsules	Pednisone	12M	(1) (2)
Sun Shaoyuan 2014	20/20	Pednisone+Shengxuexiaoban Capsules	Pednisone	16W	(1) (4) (5)
Wang Wei 2008	30/30	Pednisone+Shengxuexiaoban Capsules	Pednisone	3-6M	(1) (2) (3) (5)
Chen Jiawei et al. 2012	23/23	Pednisone+Shengxuexiaoban Capsules	Pednisone	12M	(1) (3)
Yang Jixiang 2016	22/26	Pednisone+Shengxuexiaoban Capsules	Pednisone	18M	(2) (5)
Zhang Weidong 2011	32/32	Pednisone+Shengxuexiaoban Capsules	Pednisone	10-24M	(1) (2) (3) (5)
Liang Jinqiu et al. 2007	40/30	Pednisone+Shengxuexiaoban Capsules	Pednisone	6M	(1) (2) (3)
Song Chunge et al. 2010	24/24	Pednisone+Shengxuexiaoban Capsules	Pednisone	3M~1Y	(1) (3) (5)
Xu Ling 2014	30/30	Pednisone+Shengxuexiaoban Capsules	Pednisone	3M	(1) (5)
Zhang Xiaowen 2015	55/55	Pednisone+Shengxuexiaoban Capsules	Pednisone	90D	(1) (4) (5)
Li Haiming et al. 2016	40/40	Pednisone+Shengxuexiaoban Capsules	Pednisone	Not Given	(1) (3)
Han Xiaoyue et al. 2008	35/35	Pednisone+Shengxuexiaoban Capsules	Pednisone	Not Given	(1) (3) (5)
Liu Ling et al. 2004	23/15	Pednisone+Shengxuexiaoban Capsules	Pednisone	2W	(1) (4) (5)
He Muqing et al. 2015	33/33	Pednisone+Shengxuexiaoban Capsules	Pednisone	Not Given	(1)
Ma Liangming et al. 2004	40/40	Pednisone+Shengxuexiaoban Capsules	Pednisone	16W	(1) (4)(5)
Liang Lijie et al. 2011	18/17	Pednisone+Shengxuexiaoban Capsules	Pednisone	16W	(1) (3) (5)
Wang Yi et al. 2006	50/50	Pednisone+Shengxuexiaoban Capsules	Pednisone	Not Given	(1) (5)

Note: T refers to Test Group with Pednisone+Shengxuexiaoban Capsules, and C refers to Control Group with Pednisone alone. D, Days; W, Weeks; M, Months and Y, Years. Outcome Indicators: (1) Effective Rate; (2) recurrence rate; (3) The number of platelets; (4) recovery time of platelets (≥100×10^9^); (5) adverse reactions

### 3.3 Risk of bias assessment for included RCTs

**Fig 2:**
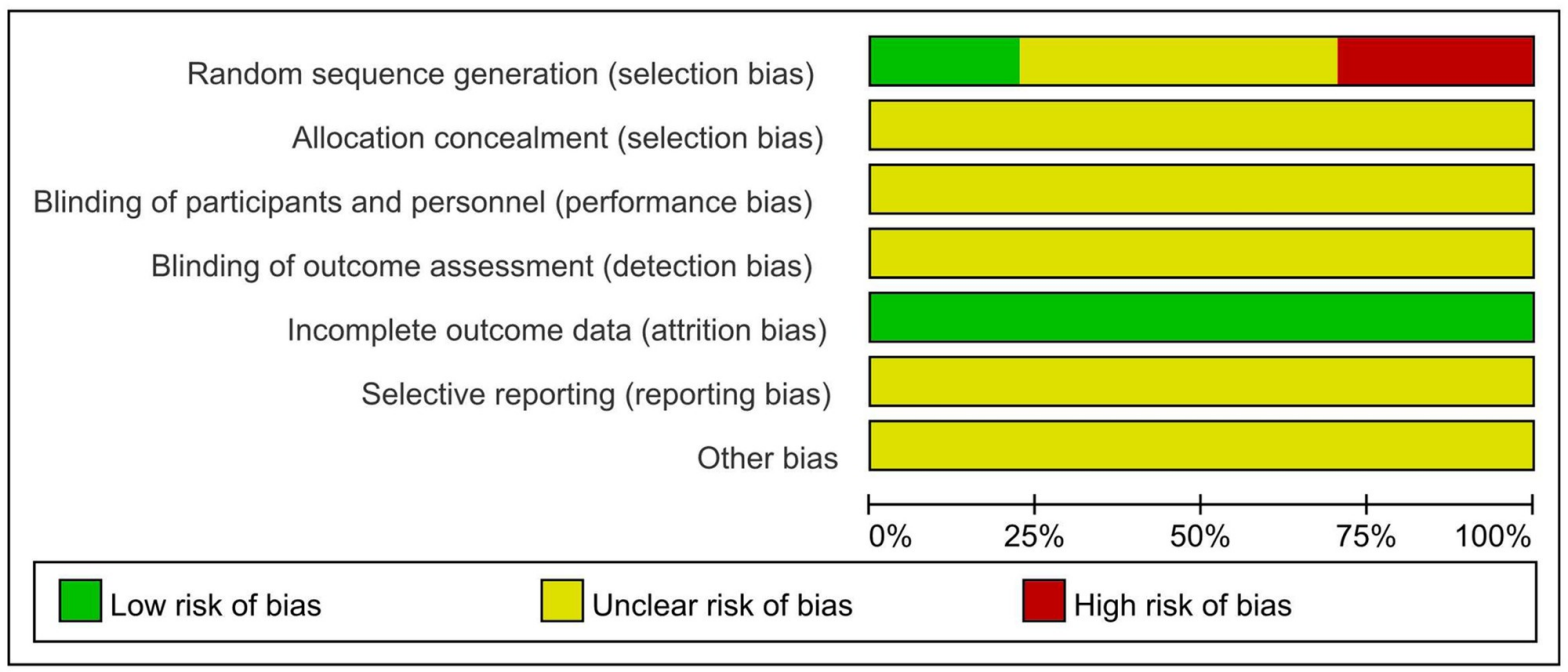
Assessment of risk of bias.

Among the 27 RCTs that were randomly grouped, 6 [5, 8–10, 16, 27] were grouped by random number table, 4 [7, 11–13] by medication regimen or therapeutic regimen, 2 [14, 15] by order of admission, and 2 [17, 21] by random odd or even number of the hospital beds (Fig 2).

### 3.4 Meta-analysis results

#### 3.4.1 Effective rate

**Fig 3:**
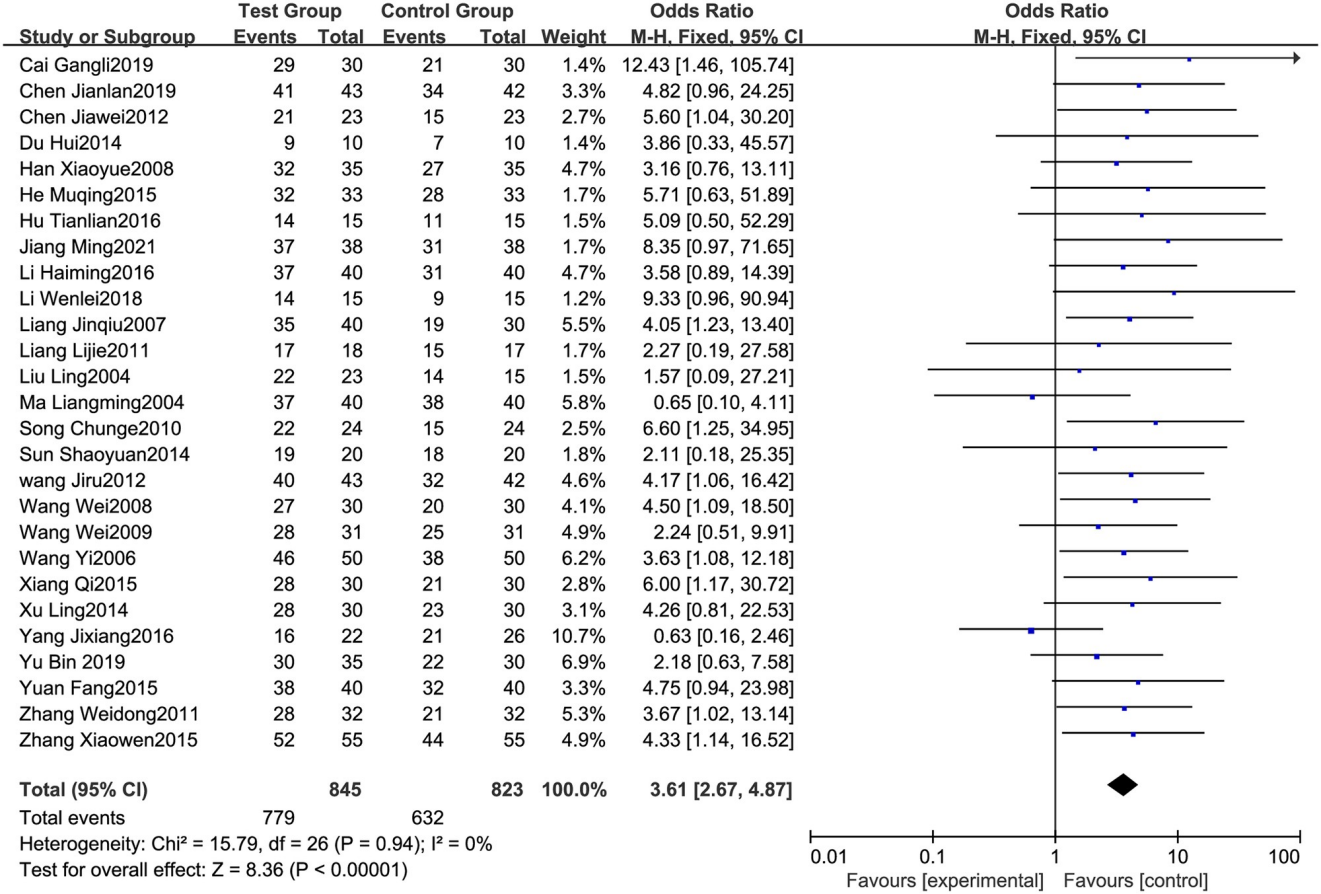
Forest plot of the effective rate.

All 27 RCTs [5–31], including 845 patients in the test group (glucocorticoid therapy combined with the Shengxuexiaoban Capsules) and 823 in the control group (glucocorticoid therapy alone), analyzed the effective rate. The heterogeneity test results were P = 0.94 and I^2^ = 0%. Therefore, the Meta-analysis used a fixed model. The results showed that the effective rate of the test group (glucocorticoid therapy combined with the Shengxuexiaoban Capsules) was higher than that of the control group (glucocorticoid therapy alone) (95% CI [2.67, 4.87], P<0.00001) (Fig 3).

#### 3.4.2 Changes in number of platelets

**Fig 4:**
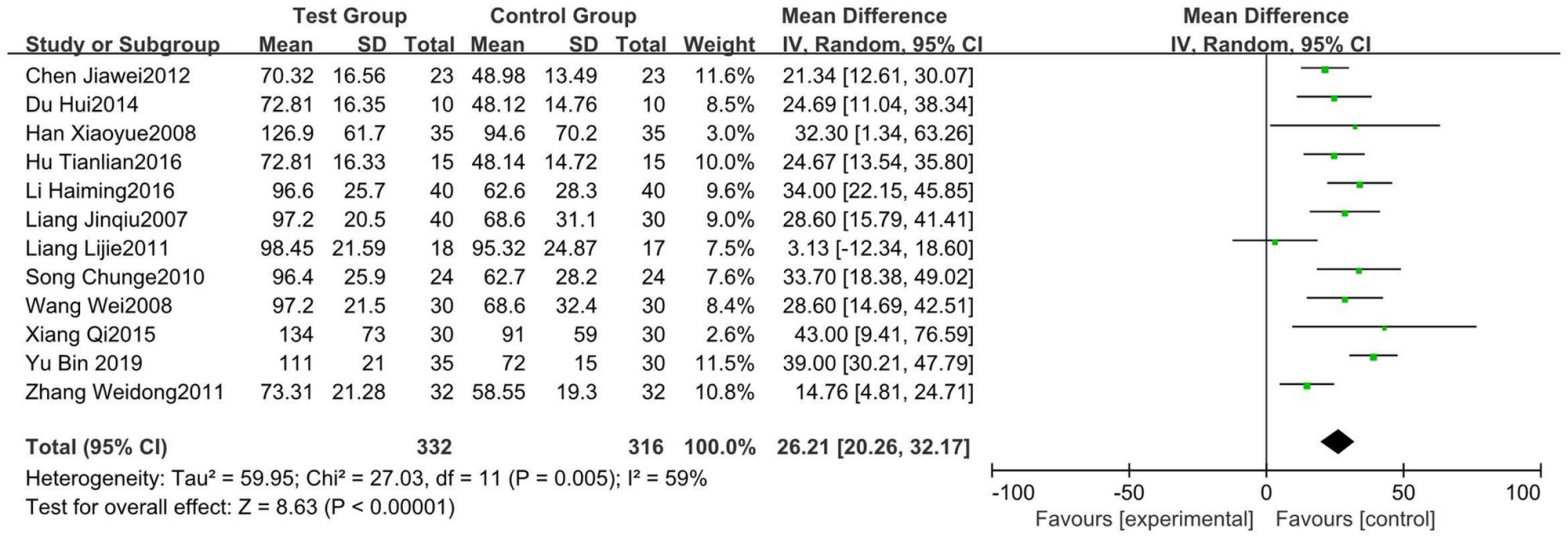
Forest plot of changes in number of platelets.

The changes in the number of platelets were measured in 12 RCTs [5, 6, 10–13, 20, 22, 25, 27–29], including 332 patients in the test group and 316 in the control group. Heterogeneity existed as the test results were P = 0.005 and I^2^ = 59%. In this case, we chose a random-effect model calculation. The results showed that the number of platelets in the test group was significantly higher than that in the control group (95%CI [20.26, 32.17], P<0.00001) (Fig 4).

#### 3.4.3 Recurrence rate

**Fig 5:**
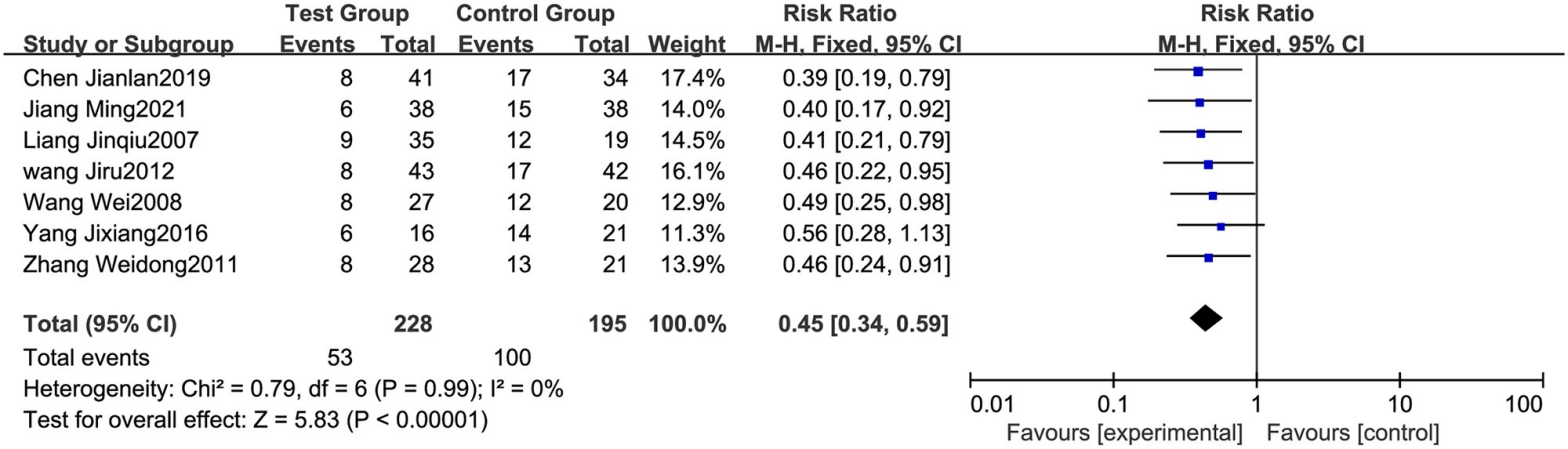
Forest plot of recurrence rate.

The recurrence rate was analyzed in seven RCTs [12, 15, 17, 21, 26–28], including 228 patients in the test group and 195 in the control group. No heterogeneity existed (the test results were P = 0.99 and I^2^ = 0%). In this case, the Meta-analysis used a fixed model. The results showed that the recurrence rate was lower when the glucocorticoid therapy was combined with the Shengxuexiaoban Capsules (95% CI [0.34, 0.59], P<0.00001) (Fig 5).

#### 3.4.4 Recovery time of platelets (≥ 100×10^9^)

**Fig 6:**
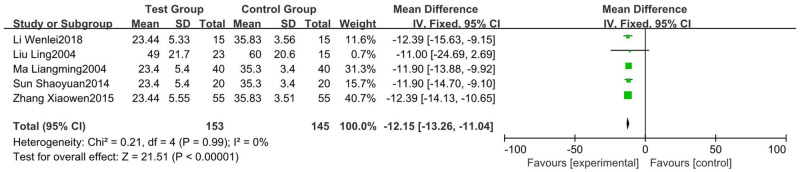
Forest plot of recovery time of platelets.

Five RCTs [16, 18, 24, 30, 31], including 153 patients in the test group and 145 in the control group, reported the time required for platelets ≥100×10^9^. No heterogeneity existed (test results were P = 0.99 and I^2^ = 0%). Therefore, we chose a random-effect model calculation. The results showed that the recovery time of platelets was shorter when the glucocorticoid therapy was combined with the Shengxuexiaoban Capsules (95% CI [-13.26, -11.04], P<0.0000) (Fig 6).

#### 3.4.5 Adverse reactions

**Fig 7:**
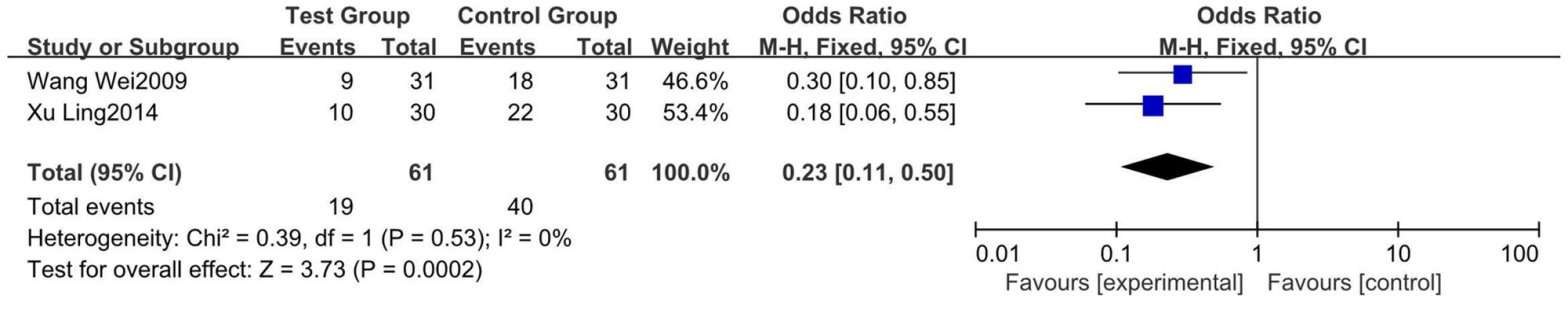
Forest plot of adverse reactions.

There were no adverse cardiac, hepatic, renal, or gastrointestinal reactions after the administration of shengxuexiaoban Capsules in any of the 16 studies [6, 7, 9, 12–16, 18, 23, 24, 26, 27, 29–31]. Four studies [6, 16, 18, 24] reported no adverse reactions in either group. Five studies [9, 14, 23, 27, 29] reported varying degrees of adverse reactions to glucocorticoid in both groups, but none affected treatment. Seven studies [7, 12, 13, 15, 26, 30, 31] did not provide specific data on the incidence of adverse reactions in the treatment and control groups. Therefore, studies [14, 23] with clearly reported adverse reactions and valid and differing data were analyzed. Heterogeneity test results: p = 0.53, I^2^ = 0%, suggesting no heterogeneity. Meta-analysis using a fixed-effects model showed that shengxuexiaoban Capsules combined with glucocorticoid therapy for ITP had fewer adverse effects than glucocorticoid therapy alone. The difference between the two groups was statistically significant (95% CI [0.11, 0.50], P = 0.0002) (Fig 7).

### 3.5 Publication bias

**Fig 8:**
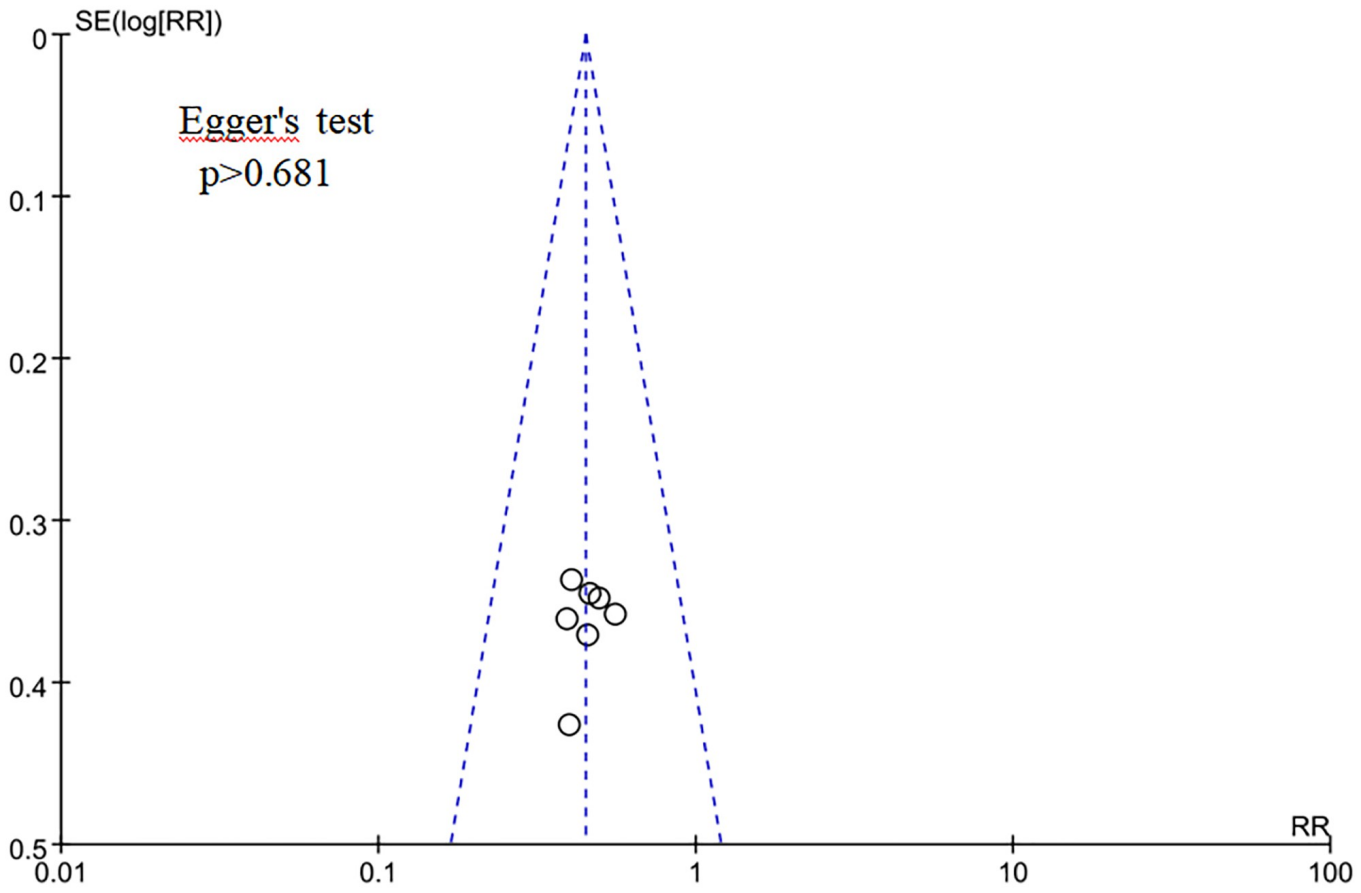
Funnel plot of recurrence rate.

Meta-analysis was performed on the above outcome indicators to evaluate the publication bias. For example, the funnel plot that represented recurrence rates was basically symmetrical (Fig 8), and Egger’s test also showed that no publication bias existed (p>0.681), suggesting that the results were reliable.

### 3.6 Evidence quality evaluation

We used the GRADE pro system to evaluate the quality of evidence for the primary outcomes: recurrence rate and effective rate. The RCT was pre-set to the highest level of evidence in the GRADE evidence quality assessment and was processed according to five degradation factors. The results suggested that the quality of the evidence in recurrence rate and effective rate were intermediate (see S3 Table in S1 File). The analysis of the included studies revealed that the main reasons for this result were poor study design and insufficient sample size.

## 4 Discussion

ITP is an autoimmune-mediated acquired disease in which immune dysfunction is the leading cause of thrombocytopenia. Humoral and cellular immunity disorders can lead to increased platelet destruction or decreased platelet production [32]. It belongs to the category of "purpura" in Chinese medicine, and blood heat is one of the primary pathogenesis of ITP [27]. Shengxuexiaoban Capsules can clear heat and detoxify the blood, cool the blood, and stop bleeding. Modern pharmacological studies have found that shengxuexiaoban capsules can significantly increase the number of platelets, significantly enhance platelet aggregation function, and significantly shorten the clotting time [10]. The results of Li Linfeng’s study [33] showed that shengxuexiaoban capsules could achieve the effect of treating ITP by suppressing the body’s immune function and reducing platelet antibody production. He Muqing [8] found that shengxuexiaoban capsules can regulate Treg cells and increase the immunosuppressive properties of Treg cells to influence the development of ITP. In addition, the glucocorticoid is a medicine that helps Yang to generate heat in Chinese medicine. Patients who take glucocorticoid for a long time may show signs of Yin deficiency and fire [34], and the effectiveness of shengxuexiaoban capsules in clearing heat and detoxifying toxins can reduce such signs.

The META analysis results showed that in treating patients with ITP, shengxuexiaoban capsules combined with glucocorticoid therapy improved the efficiency and reduced the relapse rate compared with glucocorticoids therapy alone. It can also improve platelet count, shorten platelet recovery time, and have a low incidence of adverse events. The above indicates that shengxuexiaoban capsules have an excellent therapeutic effect on ITP patients. Thus, we concluded that the effect of shengxuexiaoban capsules combined with glucocorticoid therapy for ITP is better than that of glucocorticoid therapy alone. The results of this study are consistent with the findings of previous studies by Huami Ye [35]. The number of RCTs included in this study and the sample size was significant, and the results were more accurate compared to the study by Huami Ye [35]. This study further evaluated the quality of evidence based on GRADE criteria, which provided a reference for clinical decision-making. However, the quality of evidence for this conclusion is not high, and we do not rule out the inclusion of future large-sample, multicenter, high-quality clinical studies to further validate this conclusion.

Our study has some strengths and limitations. Firstly the quality of the included RCTs was not high. Only 6 out of 27 RCTs were grouped with prednisone by a random number table, which may affect the reliability of the conclusions. Therefore more high-quality clinical studies are needed to validate the effectiveness of glucocorticoid therapy combined with Shengxuexiaoban capsules in ITP. Secondly, all RCTs used glucocorticoid therapy combined with Shengxuexiaoban capsules. However, the dose and age of Shengxuexiaoban capsules and glucocorticoid were not uniform, which may also affect the accuracy of the conclusions. However, we included a more significant number of studies than before, increased data than before, and obtained more accurate results.

In conclusion, based on the current evidence, shengxuexiaoban capsules combined with glucocorticoid are effective in treating ITP without serious adverse effects. However, due to the low quality of the included literature and the generally low quality of the evidence, more and higher quality studies are needed to validate the results and better guide the clinical practice.

## Supporting information

S1 Checklist PRISMA 2020 checklist. (DOCX)

S1 File Search strategy、 table—Risk of bias (quality) assessment table、 GRADE (certainty) assessment table. (DOCX)

S1 Table Detailed data. (XLSX)

S1 Fig PRISMA 2020 flow diagram for new systematic reviews which included searches of databases and registers only. (DOCX)
