# Supplementary material for: Effectiveness of shengxuexiaoban capsules combined with glucocorticoid therapy for immune thrombocytopenia: A meta-analysis
Source: PLoS One. 2022 Sep 30;17(9):e0275122. doi: 10.1371/journal.pone.0275122 (PMC9524648; doi:10.1371/journal.pone.0275122)
Supplement: S1 File — (DOCX) [file pone.0275122.s002.docx]

**Effectiveness of Shengxuexiaoban Capsules Combined with Hormone Therapy for Immune Thrombocytopenia: A Meta-analysis**

**1、Search strategy**

#1 免疫性血小板减少症 OR 特发性血小板减少性紫癜 OR 免疫性血小板减少性紫癜 OR 紫癜 血小板减少

#2 升血小板胶囊

#3 泼尼松 OR 强的松 OR 激素

#1 AND #2 AND #3

#1 Search (("Immune Thrombocytopenia"[Mesh]) OR（Idiopathic Thrombocytopenia purpura) OR Immune Thrombocytopenic Purpura OR Purpura thrombocytopenia;

#2 Search (Shengxuexiaoban Capsules) ;

#3 Search (glucocorticoid) OR Prednisone ;

#1 AND #2 AND #3.

**S2 Table - Risk of bias (quality) assessment table**

| ***Risk of Bias*** | **Random sequence generation (selection bias)** | **Allocation concealment (selection bias)** | **Blinding of participants and personnel (performance bias)** | **Blinding of outcome assessment (detection bias)** | **Incomplete outcome of data (attrition bias)** | **Selection reporting (reporting bias)** | **Other bias** |
| --- | --- | --- | --- | --- | --- | --- | --- |
| li2018 | Unclear risk | Unclear risk | Unclear risk | Unclear risk | Low risk | Unclear risk | Unclear risk |
| hu2016 | High risk | Unclear risk | Unclear risk | Unclear risk | Low risk | Unclear risk | Unclear risk |
| cai2019 | Unclear risk | Unclear risk | Unclear risk | Unclear risk | Low risk | Unclear risk | Unclear risk |
| yuan2015 | Low risk | Unclear risk | Unclear risk | Unclear risk | Low risk | Unclear risk | Unclear risk |
| yu2019 | Unclear risk | Unclear risk | Unclear risk | Unclear risk | Low risk | Unclear risk | Unclear risk |
| du2014 | Low risk | Unclear risk | Unclear risk | Unclear risk | Low risk | Unclear risk | Unclear risk |
| xiang2015 | Unclear risk | Unclear risk | Unclear risk | Unclear risk | Low risk | Unclear risk | Unclear risk |
| wang2012 | High risk | Unclear risk | Unclear risk | Unclear risk | Low risk | Unclear risk | Unclear risk |
| chen2019 | High risk | Unclear risk | Unclear risk | Unclear risk | Low risk | Unclear risk | Unclear risk |
| sun2014 | Unclear risk | Unclear risk | Unclear risk | Unclear risk | Low risk | Unclear risk | Unclear risk |
| wang2008 | High risk | Unclear risk | Unclear risk | Unclear risk | Low risk | Unclear risk | Unclear risk |
| chen2012 | Unclear risk | Unclear risk | Unclear risk | Unclear risk | Low risk | Unclear risk | Unclear risk |
| yang2016 | Unclear risk | Unclear risk | Unclear risk | Unclear risk | Low risk | Unclear risk | Unclear risk |
| zhang2011 | Low risk | Unclear risk | Unclear risk | Unclear risk | Low risk | Unclear risk | Unclear risk |
| liang2007 | Unclear risk | Unclear risk | Unclear risk | Unclear risk | Low risk | Unclear risk | Unclear risk |
| song2010 | Unclear risk | Unclear risk | Unclear risk | Unclear risk | Low risk | Unclear risk | Unclear risk |
| xu2014 | High risk | Unclear risk | Unclear risk | Unclear risk | Low risk | Unclear risk | Unclear risk |
| zhang2015 | Low risk | Unclear risk | Unclear risk | Unclear risk | Low risk | Unclear risk | Unclear risk |
| li2016 | Low risk | Unclear risk | Unclear risk | Unclear risk | Low risk | Unclear risk | Unclear risk |
| han2008 | Unclear risk | Unclear risk | Unclear risk | Unclear risk | Low risk | Unclear risk | Unclear risk |
| liu2004 | Unclear risk | Unclear risk | Unclear risk | Unclear risk | Low risk | Unclear risk | Unclear risk |
| wang2006 | High risk | Unclear risk | Unclear risk | Unclear risk | Low risk | Unclear risk | Unclear risk |
| liang2011 | High risk | Unclear risk | Unclear risk | Unclear risk | Low risk | Unclear risk | Unclear risk |
| ma2004 | Unclear risk | Unclear risk | Unclear risk | Unclear risk | Low risk | Unclear risk | Unclear risk |
| he2015 | Low risk | Unclear risk | Unclear risk | Unclear risk | Low risk | Unclear risk | Unclear risk |
| jiang2021 | High risk | Unclear risk | Unclear risk | Unclear risk | Low risk | Unclear risk | Unclear risk |
| wang2009 | Unclear risk | Unclear risk | Unclear risk | Unclear risk | Low risk | Unclear risk | Unclear risk |

**S3 Table- GRADE (certainty) assessment table**

| **Quality assessment** | | | | | | | **No of patients** | | **Effect** | | **Quality** | **Importance** |
| --- | --- | --- | --- | --- | --- | --- | --- | --- | --- | --- | --- | --- |
|  |  |  |  |  |  |  |  |  |  |  |  |  |
| **No of studies** | **Design** | **Risk of bias** | **Inconsistency** | **Indirectness** | **Imprecision** | **Other considerations** | **Shengxuexiaobanjiaonang for ITP** | **Control** | **Relative (95% CI)** | **Absolute** |  |  |
| **relapse** | | | | | | | | | | | | |
| 7 | randomised trials | serious^1^ | no serious inconsistency | no serious indirectness | no serious imprecision | none | 53/228  (23.2%) | 100/195  (51.3%) | RR 0.45 (0.34 to 0.59) | 282 fewer per 1000 (from 210 fewer to 338 fewer) | ⊕⊕⊕O MODERATE | CRITICAL |
|  |  |  |  |  |  |  |  | 60% |  | 330 fewer per 1000 (from 246 fewer to 396 fewer) |  |  |
| **the effective rate** | | | | | | | | | | | | |
| 27 | randomised trials | serious^1^ | no serious inconsistency | no serious indirectness | no serious imprecision | none | 779/845  (92.2%) | 632/823  (76.8%) | OR 3.61 (2.67 to 4.87) | 155 more per 1000 (from 130 more to 174 more) | ⊕⊕⊕O MODERATE | CRITICAL |
|  |  |  |  |  |  |  |  | 76.7% |  | 155 more per 1000 (from 131 more to 174 more) |  |  |
| **the recovery time of platelets (≥ 100×109) (Better indicated by lower values)** | | | | | | | | | | | | |
| 5 | randomised trials | serious^1^ | serious | no serious indirectness | no serious imprecision | none^2^ | 153 | 145 | - | MD 12.15 lower (13.26 to 11.04 lower) | ⊕⊕OO LOW | IMPORTANT |
| **Changes in Number of Platelets (Better indicated by lower values)** | | | | | | | | | | | | |
| 12 | randomised trials | serious^3^ | no serious inconsistency | no serious indirectness | no serious imprecision | reporting bias | 332 | 316 | - | MD 26.21 higher (20.26 to 32.17 higher) | ⊕⊕OO LOW | IMPORTANT |

^1^ Random,blinded risk of bias
^2^ There is publication bias
^3^ Insufficient sample size
