# Supplementary material for: Effectiveness of shengxuexiaoban capsules combined with glucocorticoid therapy for immune thrombocytopenia: A meta-analysis
Source: PLoS One. 2022 Sep 30;17(9):e0275122. doi: 10.1371/journal.pone.0275122 (PMC9524648; doi:10.1371/journal.pone.0275122)
Supplement: S1 Fig — (DOCX) [file pone.0275122.s004.docx]

**Identification of studies via databases and registers**

Records removed *before screening*:

Duplicate records removed (n = 75)

Records marked as ineligible by automation tools (n =0 )

Records removed for other reasons (n = 0)

Records identified from Chinese and English databases*:

Databases (n =172 )

Registers (n =0 )

**Identification**

Records screened

(n = 97)

Records excluded ( recording meetings, animal experiments, irrelevant articles)**

(n = 66)

Reports sought for retrieval

(n =31 )

Reports not retrieved

(n = 0)

**Screening**

Reports assessed for eligibility

(n =31)

Reports excluded:

Articles that do not fit the research design (n =4 )

Studies included in review

(n =27 )

Reports of included studies

(n =27 )

**Included**

*Consider, if feasible to do so, reporting the number of records identified from each database or register searched (rather than the total number across all databases/registers).

**If automation tools were used, indicate how many records were excluded by a human and how many were excluded by automation tools.

*From:*  Page MJ, McKenzie JE, Bossuyt PM, Boutron I, Hoffmann TC, Mulrow CD, et al. The PRISMA 2020 statement: an updated guideline for reporting systematic reviews. BMJ 2021;372:n71. doi: 10.1136/bmj.n71

For more information, visit: <http://www.prisma-statement.org/>
